# Setaria Comes of Age: Meeting Report on the Second International Setaria Genetics Conference

**DOI:** 10.3389/fpls.2017.01562

**Published:** 2017-09-28

**Authors:** Chuanmei Zhu, Jiani Yang, Christine Shyu

**Affiliations:** Donald Danforth Plant Science Center, St. Louis MO, United States

**Keywords:** Setaria, genetic model, agronomically important traits, drought tolerance, inflorescence architecture, C_4_ photosynthesis, millet, translational research

## Abstract

*Setaria viridis* is an emerging model for cereal and bioenergy grasses because of its short stature, rapid life cycle and expanding genetic and genomic toolkits. Its close phylogenetic relationship with economically important crops such as maize and sorghum positions Setaria as an ideal model system for accelerating discovery and characterization of crop genes that control agronomically important traits. The Second International Setaria Genetics Conference was held on March 6–8, 2017 at the Donald Danforth Plant Science Center, St. Louis, MO, United States to discuss recent technological breakthroughs and research directions in Setaria (presentation abstracts can be downloaded at https://www.brutnelllab.org/setaria). Here, we highlight topics presented in the conference including inflorescence architecture, C_4_ photosynthesis and abiotic stress. Genetic and genomic toolsets including germplasm, mutant populations, transformation and gene editing technologies are also discussed. Since the last meeting in 2014, the Setaria community has matured greatly in the quality of research being conducted. Outreach and increased communication with maize and other plant communities will allow broader adoption of Setaria as a model system to translate fundamental discovery research to crop improvement.

## Introduction

**FIGURE 1:**
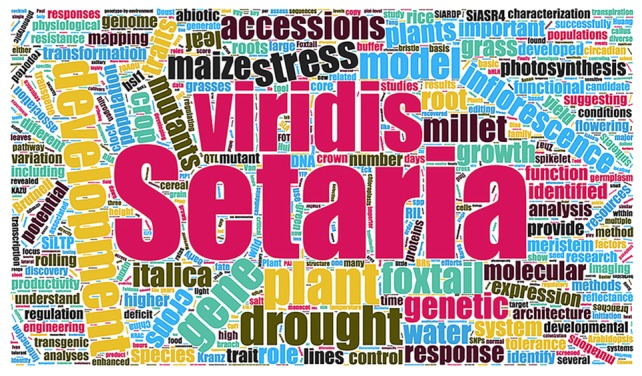
Word cloud of abstracts presented in the Second International Setaria Genetics Conference generated using http://www.wordclouds.com/.

**FIGURE 2:**
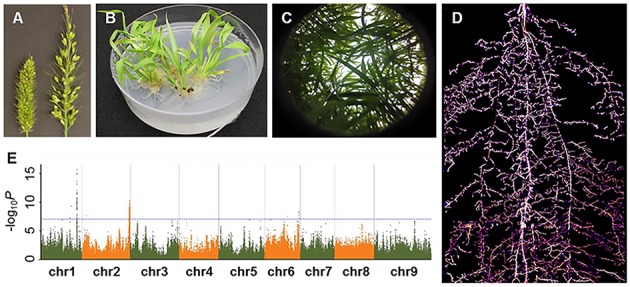
A glimpse of Setaria research presented in the Second International Setaria Genetics Conference. **(A)** Forward genetic mutant screens. Inflorescence mutants (right panicle) isolated from a chemical-induced mutant screen (Photo courtesy of Hui Jiang). **(B)** Callus-based transformation in *S. viridis* (Photo courtesy of Veena Veena). **(C)** Above ground phenotyping. Hemispherical image of *S. viridis* shoots in field settings (Photo courtesy of Darshi Banan and Andrew Leaky). **(D)** Below ground phenotyping. *S. viridis* root system at 21 days after planting using Growth and Luminescence Observatory for Roots (GLO-Roots) (Photo courtesy of Jose Sebastian and José Dinneny). **(E)** Genome wide associate study in *S. italica*. Horizontal and vertical axes denote genomic position (in each chromosome) and –log_10_ *P* for all SNPs, respectively (Photo courtesy of Guanqing Jia).

The use of *Setaria italica* as a model system for cereal and bioenergy grasses was proposed nearly a decade ago (Doust et al., 2009) and because of its short stature, rapid life cycle and ability to be transformed, the proposal to focus on its wild ancestor, *Setaria viridis*, soon followed (Brutnell et al., 2010). Since then, the genome of *S. italica* has been sequenced (Bennetzen et al., 2012), transformation technologies have been improved for *S. viridis* (Van Eck and Swartwood, 2015) and extensive germplasm collections have been generated for both species (Jia et al., 2013; Huang et al., 2014). The close phylogenetic relationship that Setaria shares with other agronomically important grasses such as maize, sorghum and sugarcane will help bridge gene discovery to engineering or breeding for improved performance in several agronomically important traits such as inflorescence architecture, C_4_ photosynthesis, and drought tolerance (Li and Brutnell, 2011; Lata et al., 2013; Huang et al., 2016; Pant et al., 2016). As an emerging model and an ancient crop, Setaria also offers tremendous opportunities to study domestication and crop improvement strategies. The First International Setaria Genetics conference was held in 2014 in Beijing, China with a focus on promoting Setaria as a model system for studying functional genomics in grasses. Research on germplasms, breeding and expression analysis were presented and the needs for transformation pipelines were discussed. The Second International Setaria Genetics Conference was held on March 6–8, 2017 at the Donald Danforth Plant Science Center (DDPSC), St. Louis, MO, United States. This conference addressed the needs discussed in the First International Setaria Genetics Conference including advancement of transformation and genome editing technology, and provided a platform for the Setaria community to present their research and discuss challenges. Here, we review highlights of the conference (**Figures 1**, **2**) and provide perspectives on the future of Setaria research.

## Meeting Report

The meeting opened with a welcome by Dr. Thomas Brutnell (DDPSC), followed by the plenary speaker Dr. Andrew Doust (Oklahoma State University). Doust provided an overview of the past, present, and future research of Setaria, highlighting advantages and opportunities of using Setaria as a genetic model to study drought resistance, flowering time, C_4_ photosynthesis, microbe interactions, and cell wall biosynthesis. Molecular and genetic toolsets including transposable element tagging populations were also discussed, with more detailed information available in the recently published book titled *Genetics and Genomics of Setaria*^1^. As an evolutionary biologist, Doust also emphasized the need to use multiple model systems, Setaria being one of them, to understand the basis for morphological diversity and genome evolution in the grasses. He also advocated Setaria as an education model due to its robust growth and small size, ideal for the classroom, to communicate science to the public.

## Plant Development

Developmental regulation of plant architecture in grasses has long been a research area of interest for its potential to improve crop yields (Mathan et al., 2016). Dr. Andrea Eveland (DDPSC) reported the characterization of *bristleless* mutants in *S. viridis*, which were identified in a forward genetics screen for altered panicle phenotypes. These mutants revealed a novel role for a class of phytohormones, brassinosteroids, in controlling inflorescence development, specifically the decision to produce spikelets or acquire the fate of sterile modified branches called bristles, characteristic of certain Setaria spp. and related grasses. Dr. Chuanmei Zhu (Kellogg lab, DDPSC) discussed work using CRISPR-Cas9 technology (reverse genetics) to pinpoint the functions of *CLE* genes in meristem formation and maintenance in *S. viridis*. In a poster presentation, Huang et al. (2017) (DDPSC) described the *aux1* mutant from *S. viridis*, which was also shown to regulate inflorescence architecture in maize. Collectively, these projects demonstrate rapid progress in both forward and reverse genetics approaches in Setaria to address basic biological questions related to inflorescence development, and the potential of translating findings to other economically important crops such as maize.

## C_4_ Photosynthesis

Molecular and genetic characterization of genes and pathways in C_4_ photosynthesis has been challenging in the past due to the lack of model systems and limited molecular tools to dissect this process. Now with rapidly emerging resources in Setaria, a C_4_ grass, the genetic basis underlying C_4_ metabolism and anatomy can be explored. Dr. Pu Huang (Brutnell Lab, DDPSC) utilized genomic resources in *S. viridis* and other grass species to identify C_4_ related candidate genes by searching for signals of adaptive evolution across multiple species in C_4_ lineages (Huang and Brutnell, 2016). Dr. Carla Coelho from the same lab presented her work to define the functions of *INDETERMINATE DOMAIN* (*IDD*) transcription factors in establishing Kranz anatomy, a unique anatomical structure of C_4_ plants. Confocal imaging techniques were utilized on both transiently and stably transformed *S. viridis* plants with IDDs tagged with fluorescent proteins. Molecular studies of genes involved in the regulation of C_4_ cell fate, such as bHLH transcription factors and chloroplast-localized RNA binding proteins, were also discussed in poster presentations. Presentations from Benson Hill Biosystems, a St. Louis startup company, further demonstrated the utilization of *S. viridis* as a model for C_4_ crop development, suggesting that studies in Setaria have strong potential for translating to crop improvement strategies.

## Abiotic Stress

Drought response was a major focus of research at the conference as Setaria is highly drought tolerant. A broad range of studies from molecular characterization of drought tolerance genes to field phenotyping of diversity panels and recombinant inbred line (RIL) populations was reported. Dr. Andrew Leakey (University of Illinois) presented bioassay and physiological measurement protocols that included measurements for leaf canopy traits, stomatal conductance, photosynthesis efficiency and water potential (**Figure 2C**). Leaf rolling phenotypes in response to drought were also characterized in greater detail as presented by Darshi Banan from the Leaky lab. In addition to above-ground phenotypes, Dr. Jose Sebastian (Dinneny Lab, Carnegie Institution for Science) discussed the role of water availability on root phenotypic variation. Specifically, drought treatment led to restricted growth of crown roots in both Setaria and maize (Sebastian et al., 2016). Reporter lines coupled with a rhizotron imaging system were used to visualize root growth phenotypes in response to drought (**Figure 2D**; Rellán-Álvarez et al., 2015). With these phenotyping methods in place, mutagenized populations are currently being screened and mutant lines can be characterized in greater detail to uncover molecular mechanisms underlying drought tolerance. In addition to drought stress, Setaria responses to heat, salt, and herbicide stress were discussed in several poster presentations though most characterizations were in combination with drought tolerance studies. Biotic stress responses were less discussed throughout the meeting, but a poster on generating mutants in the jasmonate signaling pathway in Setaria indicates that interest in studying herbivore and pathogen resistance is growing in the community.

## Germplasm, Population Genetics and Genomics

Genomic resources and population genetics studies presented at the conference highlighted the breadth of Setaria resources and demonstrated how emerging molecular tools and resources can be nicely complemented. *S. viridis* is the most widely distributed weed in the world while *S. italica* is one of the most ancient domesticated crops in China (Lu et al., 2009). Both have great natural diversity and provide important resources for novel gene/allele mining for agronomically important traits. Dr. Guanqing Jia (Chinese Academy of Agricultural Sciences, China) presented his work on population genetics and genome wide association studies (GWAS) in *S. italica*. His group has sequenced 916 diverse accessions of *S. italica* and has identified 512 loci associated with 47 agronomically important traits including flowering time, plant height and inflorescence architecture (Jia et al., 2013). This study provides many candidate genes/loci for functional validation that can be performed in Setaria. Dr. Hari Upadhyaya [International Crops Research Institute for the Semi-Arid Tropics (ICRISAT)] shared information on ICRISAT’s collection of six mandate crops, including a core collection of 155 *S. italica* accessions that are available for worldwide distribution. In addition to *S. italica* populations, *S. viridis* diversity panels have also been assembled from samples harvested across the United States (Huang et al., 2014). Together, these collections offer new opportunities for trait discovery and domestication studies.

Chemically induced mutant populations of *S. viridis* and *S. italica* have also been generated in the Brutnell and Diao Labs (DDPSC and Chinese Academy of Agricultural Sciences, respectively), providing useful materials for genetic studies in Setaria. Protocols for mapped-based cloning and bulked segregant analysis (BSA) are now available for discovery of causal genes in a time-efficient and cost-effective manner. Using these methods, *AUX1*, a gene regulating inflorescence architecture, has been identified in *S. viridis* (Huang et al., 2017). Similarly, Dr. Sha Tang (Chinese Academy of Agricultural Sciences, China) reported using this method to clone genes in *S. italica* that control grain size. A transposon tagging population in *S. viridis* is available from the Brutnell Lab at DDPSC (Kikuchi et al., 2017), strengthening resources for both forward and reverse genetic studies.

## Transformation and Genome Editing

A key prerequisite for any model system to be widely adopted is the breadth and depth of its technological toolbox. Therefore, it is not surprising that a highlight of the conference was the session on transformation and genome editing. Successful transformation in *S. viridis* was reported in 2010 (Brutnell et al., 2010). However, until recently, transformation efficiencies have been low with tissue culture-based agrobacterium methods and this has been rate limiting for molecular analyses to accelerate. Dr. Joyce Van Eck (Boyce Thompson Institute) summarized recent advances and resources available for *S. viridis* transformation. These include identification and utilization of a new accession, ME034V that is morphologically similar to A10.1 but exhibits significantly higher transformation efficiency (up to 80%). Transformation pipelines for both A10.1 and ME034V are now performed routinely and available to the community at the Boyce Thompson Institute and Donald Danforth Plant Science Center. Van Eck is also working to develop the *Baby boom*/*Wuschel* system (Lowe et al., 2016) for Setaria, which is anticipated to greatly reduce time and labor for transformation procedures. Recent publications reporting spike dip transformation in *S. viridis* are promising but several independent research groups still struggle to reproduce these results (Martins et al., 2015; Saha and Blumwald, 2016). This is potentially due to variation in environment and/or germplasm among labs. Nevertheless, the promise of floral dip suggests that a coordinated and sustained effort across multiple labs could potentially have a significant return.

Another notable highlight from the conference was the successful use of CRISPR-Cas9 technology (Demirci et al., 2017) to create allelic variants in *S. viridis*. Multiple research groups have now targeted genes of interest at high editing efficiencies and have observed phenotypes from CRISPR lines at as early as the T_0_ stage. Presentations from Drs. Dan Voytas (University of Minnesota) and Emma January (Benson Hill Biosystems) highlighted key advances in genome editing technology such as vector toolkits for multiplexed genome editing, homologous recombination, utilizing RNA viruses as well as precise insertions utilizing novel nucleases (Čermák et al., 2017). It is exciting to see how the field is rapidly developing as both academic and industry groups are designing and testing these technologies in Setaria.

## Perspectives

The development of rich genetic and genomic resources for Setaria over the last few years have made Setaria an excellent genetic model to study fundamental and applied questions in grasses such as C_4_ photosynthesis, developmental biology and biotic and abiotic stress tolerance. Research in Setaria will enable strategies for breeding enhanced productivity, not only in economically important crops such as maize and sorghum, but also in “orphan” crops such as fonio (acha), and pearl, finger, and foxtail millets that are staples in the semiarid tropics of Asia and Africa but are often neglected by major breeding efforts.

Since the last Setaria meeting in 2014 in Beijing, China, it was clear that the community has matured greatly in the quality of research, however, it has not grown much in number. Dr. Jeff Bennetzen (University of Georgia) gave the closing remarks and encouraged the Setaria community to reach out to the maize and other related communities to inform researchers of the emerging resources available for Setaria and how these can advance their works. Specific needs for the community were also discussed. For example, quite a bit of genomics and transcriptomics data have been generated but few metabolomic, proteomic and epigenetic studies have been conducted, providing exciting opportunities for researchers with these expertise. As datasets continue to accumulate, the need has increased for a centralized resource interface to access these datasets. In addition, despite Setaria being amenable to tissue culture based transformation, a robust floral dip method would greatly accelerate broader adoption. Together, with the commitment of current members and the addition of new blood, growth and maturation of the Setaria community and the research in Setaria is highly anticipated.

## Author Contributions

CS, CZ, and JY conceived of and wrote the manuscript. All authors read and approved the final manuscript.

## Conflict of Interest Statement

The authors declare that the research was conducted in the absence of any commercial or financial relationships that could be construed as a potential conflict of interest.

## Footnotes

1 http://www.springer.com/us/book/9783319451039
